# Time- and Dose-Dependent Cardiovascular Effects of Nicotine-Containing Electronic Cigarettes in Young Adults: A Systematic Review and Meta-Analysis

**DOI:** 10.3390/toxics13100831

**Published:** 2025-09-30

**Authors:** Carmen Ranchal-Lavela, David Casanova-Rodríguez, Antonio Ranchal-Sanchez, María José De La Torre-Aguilar, Jose Manuel Jurado-Castro

**Affiliations:** 1 Department of Nursing, Pharmacology and Physiotherapy, Faculty of Medicine and Nursing, University of Cordoba, 14004 Cordoba, Spain; h92ralac@uco.es (C.R.-L.); davidcasanovafisio@gmail.com (D.C.-R.); 2 Maimonides Biomedical Research Institute of Cordoba (IMIBIC), Reina Sofia University Hospital, University of Cordoba, 14004 Cordoba, Spain; delatorremj4@gmail.com (M.J.D.L.T.-A.); juradox@gmail.com (J.M.J.-C.); 3 CIBER Fisiopatología de la Obesidad y Nutrición (CIBEROBN), Instituto de Salud Carlos III, 28029 Madrid, Spain; 4 Ciencias de la Actividad Física y El Deporte, Escuela Universitaria de Osuna (Centro Adscrito a la Universidad de Sevilla), 41640 Osuna, Spain

**Keywords:** electronic nicotine delivery systems, young adults, nicotine, cardiovascular effects, blood pressure, meta-analysis

## Abstract

Objective: To synthesize the current evidence on the cardiovascular effects of electronic cigarettes (ECs) in young adults (18–30 years), distinguishing between acute and chronic exposure, and comparing their effects to conventional tobacco (CT) use. Methods: A systematic review and meta-analysis (PROSPERO: CRD420251072847) was conducted following PRISMA guidelines. A total of 21 studies (12 RCTs, 8 case–control, 1 cohort) involving 17241 participants were included. Results: Acute EC use, particularly with nicotine, significantly increased systolic blood pressure (SBP: MD = 3.14 mmHg, 95% CI: 0.76 to 5.52), diastolic blood pressure (DBP: MD = 2.05 mmHg, 95% CI: 0.85 to 3.25), and heart rate (HR: MD = 4.23 bpm, 95% CI: 2.10 to 6.37), with effects most pronounced at 0 min post-exposure and dissipating within 1 h. Chronic EC use was associated with reduced endothelium-dependent vasodilation and early atherosclerotic changes. Nicotine-free ECs induced fewer cardiovascular alterations. Comparisons with CT revealed less severe cardiovascular damage with ECs, though still significant when compared to non-smokers. Conclusion: Nicotine-containing EC use in young individuals is associated with modest, predominantly acute and dose-dependent, cardiovascular effects, including transient increases in BP and HR. While initially less harmful than CT, the evidence is largely from cross-sectional studies and acute use, so ECs cannot be considered safe and their use warrants caution in youth.

## 1. Introduction

Electronic cigarettes (ECs) are devices classified within the group known as “electronic nicotine delivery systems” (ENDSs). Their mechanism of action is based on heating a liquid to generate an inhalable aerosol. This liquid typically contains nicotine, flavoring agents, and either propylene glycol or glycerin [1]. ECs are composed of two primary components [2], i.e., a “mod”, which is the battery that supplies the electrical current, and an atomizer, where the liquid is stored and vaporized upon contact with the current. The resulting aerosol has a white appearance similar to water vapor, which is why these devices are commonly referred to as “vapes”. It is important to distinguish ECs from so-called “heated tobacco products”, which, although they also generate aerosols, do so by heating processed tobacco leaves at high temperatures rather than by vaporizing a liquid solution [3].

ECs, with or without nicotine, were initially designed as substitutes for conventional combustion tobacco (CT) products, simulating their mode of use through inhalation and vapor formation, with the aim of serving as a less harmful alternative to support smoking cessation [4]. In fact, in such countries as the United Kingdom, ECs are one of the tools employed to aid smoking cessation. Some authors have reported greater effectiveness of ECs compared to nicotine replacement therapy in adults, provided their use is accompanied by behavioral support [5]. However, although several studies have demonstrated that the levels of toxicants and carcinogens in ECs are lower than those found in CT products, current knowledge regarding the potential health effects of inhaling the aerosol generated from e-liquids remains limited [6].

Most studies conducted to date on EC toxicity have compared with the harms associated with tobacco combustion, a process that differs fundamentally from the one used by electronic devices. In fact, CO-oximetry measurements in EC users show levels of exhaled carbon monoxide comparable to those of non-smokers.

On the other hand, the recent introduction of these products to the market, combined with intensive advertising on the Internet and promotion by social media influencers, has facilitated their widespread reach, particularly among adolescents and young adults. This demographic group is a primary target for the tobacco industry due to their potential for long-term use, thereby increasing the risk of smoking initiation within this age group [7]. According to the Spanish “ESTUDES 2023” survey, more than half of students aged 14 to 18 (54.6%) admit to having used e-cigarettes at least once in their lives. This represents an increase of 10.3 percentage points compared to 2021, placing the use of these devices at its highest point in the historical series [8]. Globally, the prevalence of EC use among young people is 16.8%, with the United States and Taiwan reporting the highest rates [9].

The growing use of ECs among youth in recent years is a concerning trend, particularly given the potential health risks and the increasing evidence that ECs are not as harmless as initially assumed [4]. In this regard, the scientific community raised alarms about the condition known as “E-cigarette or Vaping-Associated Lung Injury” (EVALI), a lung disease linked to EC use that has resulted in numerous deaths among young individuals in the United States [10]. Therefore, the exposure of young people to these devices is especially troubling, not only because of their increased susceptibility to the addictive potential of nicotine, but also due to the toxic potential of other substances contained in these products, such as propylene glycol, glycerin, various flavorings and scents, volatile organic compounds, like benzene, heavy metals, and other chemicals that have already been associated with damage to the respiratory and cardiovascular systems [11]. Most studies on the effects of components, such as glycerol, flavorings, or polyethylene glycol in the aerosol, have focused on oral, respiratory, or dermal exposure in humans. The potential harmful effects resulting specifically from the inhalation of these substances remain unknown [4]. Siddiqi et al. (2023) [12] showed in their meta-analysis that ECS is associated with an increase in cardiovascular hemodynamic measures and biomarkers, although it was conducted in a general population neither focused on young people nor on time-dependent effects.

Therefore, the primary objective of this systematic review and meta-analysis was to synthesize the most recent evidence on the cardiovascular effects of EC use in young populations. Specifically, the study sought to evaluate whether these effects are more consistently observed after acute or chronic exposure, and to compare the magnitude of cardiovascular effects associated with ECs. An additional aim was to explore the potential influence of nicotine content on acute cardiovascular effects in EC users and the time- and dose-dependent cardiovascular effects.

## 2. Materials and Methods

### 2.1. Design

A systematic review and meta-analysis was conducted following the recommendation from the Preferred Reported Items for Systematic Review and Meta-analysis (PRISMA) statement [13]. The systematic review protocol was registered in PROSPERO (International Prospective Register of Systematic Reviews), with the registration number CRD420251072847.

### 2.2. Inclusion and Exclusion Criteria

The inclusion criteria for the studies were defined according to the Population, Intervention, Comparator, Outcome, and Study Design (PICOs) framework [14]. Accordingly, the population (P) consisted of young adults, and studies were included if the mean age of participants ranged between 18 and 30 years, regardless of whether some individual participants fell outside this range; the intervention (I) involved either acute or chronic exposure to nicotine-containing ENDSs; the comparator (C) included non-smoking young adults or young smokers using CT or non-nicotine ENDSs; the outcome (O) referred to cardiovascular effects resulting from such exposure; and the study design (S) comprised cross-sectional studies, cohort studies or crossover interventions.

The exclusion criteria applied were as follows: (1) studies conducted in animal models; (2) studies analyzing the effectiveness of strategies aimed at promoting cessation of ENDS use; (3) studies addressing marketing and/or advertising strategies related to ENDS consumption; (4) qualitative studies focused on perceptions, usage patterns, associated behaviors, or consumption patterns of ENDSs; (5) case reports, systematic reviews or meta-analysis; (6) studies examining heated tobacco products rather than ENDSs; (7) studies comparing different types of ENDSs without analyzing their harmful effects.

### 2.3. Search Strategy

The search was conducted by two independent researchers (CRL and DCR) in the databases Medline (PudMed), Scopus, and Web of Science (WoS) until June of 2025. The search was carried out by combining the mesh terms “electronic nicotine delivery system”, “young adults”, “health”, or “prevalence” with Boolean operators (Supplementary Material S1), and the Rayyan web app [15] was used throughout the screening phase before the actual review process.

### 2.4. Selection Criteria and Data Extraction

The review process consisted of three individual phases, namely a screening of the databases, a review of the databases, and data extraction.

In the screening phase, two individual screeners (CRL and DCR) performed the initial analysis of study titles and abstract. If the studies fit the inclusion criteria and were relevant to the review or no agreement was achieved in this phase, the full text was reviewed.

In the review phase, the full text was assessed to determine whether it met the inclusion criteria by the same screeners as the first phase. If after the full text assessment any discrepancies existed, they were resolved by consensus with a third reviewer (ARS) if necessary [13].

Finally, the data extraction phase was conducted to extract any essential information from the selected studies. Data from the included studies were systematically extracted and tabulated using a standardized table capturing the following information: author and year of publication, study design, study groups, sample size, mean age, sex distribution (female/male), nicotine concentration in electronic cigarettes (mg/mL), measured effects, assessed variables, and main results. Specifically, data on blood pressure and heart rate were extracted for subsequent meta-analysis.

Inter-rater agreement was assessed using Cohen’s kappa coefficient [16] and was interpreted as follows: negligible if <0.2, fair if 0.21–0.4, moderate if 0.41–0.6, substantial if 0.61–0.8, and almost perfect if 0.81–1, according to the criteria described by Landis and Koch (1977) [17].

### 2.5. Quality and Risk of Bias Evaluation

Firstly, to evaluate the quality of the included studies two independent tools were used, namely the STROBE declaration for the observational studies and the CONSORT declaration for the crossover interventions.

Secondly, the risk of bias of the included crossover interventions was assessed with the Cochrane Risk of Bias 2 (RoB 2) [18] tool. This tool evaluates bias derived from the randomization process, deviation from intended intervention, missing outcome data, measurement of the outcome, and selection of the reported results, which were evaluated as having a low risk of bias, some concerns, or a high risk of bias.

### 2.6. Statistical Analysis

All statistical analyses were conducted using Review Manager (RevMan) version 5.4.1, following a random effects model to account for potential between-study heterogeneity. Continuous outcomes were summarized using mean differences (MDs) and 95% confidence intervals (CIs). Separate meta-analyses were performed for systolic blood pressure (SBP), diastolic blood pressure (DBP), and heart rate (HR), comparing nicotine exposure with nicotine-free conditions. When available, subgroup analyses were conducted based on the timing of post-exposure assessment (e.g., 0 min, 30 min, and ≥1 h) and nicotine concentration (e.g., 3 mg/mL, 18 mg/mL).

Statistical heterogeneity was assessed using the chi-squared test (χ^2^) and quantified with the I^2^ statistic, with values of 25%, 50%, and 75% representing low, moderate, and high heterogeneity, respectively [19]. A significance level of *p* < 0.05 was considered for all comparisons. Where applicable, differences between subgroups were tested to explore time-dependent effects and nicotine-dependent effects.

## 3. Results

### 3.1. Studies Selected

**Figure 1:**
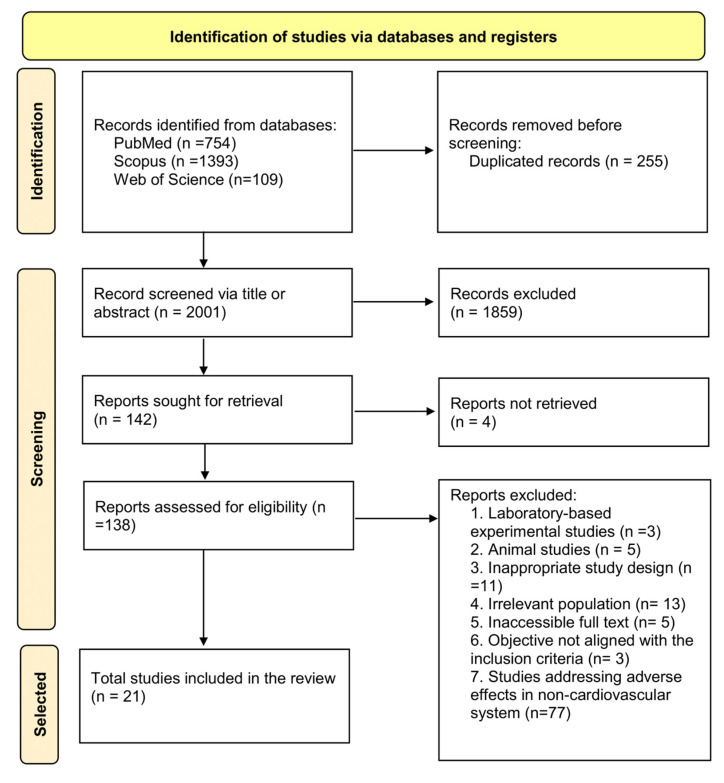
Flowchart for systematic reviews recommended by PRISMA 2020 [13].

A total of 2256 records were identified through the initial database search, of which 255 were removed as duplicates. The remaining 2001 records were screened based on title and abstract, resulting in the exclusion of 1859 records. Subsequently, 142 full-text articles were sought for retrieval, but 4 could not be obtained. A total of 138 full-text articles were assessed for eligibility, and 21 studies met the inclusion criteria. These included 12 crossover interventions, 8 cross-sectional studies, and 1 cohort study (Figure 1). Inter-rater agreement for the inclusion of articles was almost perfect, with a Cohen’s kappa coefficient of 0.9, indicating a high level of consistency between reviewers.

### 3.2. Description and Characteristics of the Studies

The total sample comprised 17,241 youths (1258 EC users including dual consumers), with a mean age ranging from 18 to 30 years. All studies were published between 2015 and 2024.

The included studies focused on regular users of ENDSs and compared them with CT users and non-smokers. The average duration of ENDS use varied across studies, ranging from approximately 3 months [20,21,22], to over 6 months [23] and more than one year [24,25,26,27,28].

Sample sizes ranged from 10 to 30 participants in most studies. An exception was the study by Shi et al., (2023) [22], a cohort study with a sample with 372 EC users, 573 dual users, 4933 CT users, and 10,486 non-smokers which investigated chronic effects, particularly hypertension, among ENDS users, CT users, and dual users. Apart from the study by Shi et al., only Kelesidis et al. (2023) [25] and Sahota et al. (2021) [21] also assessed chronic health effects, whereas the remaining studies primarily focused on acute outcomes (Table 1). Specific characteristics about the study design of every individual study are presented in Supplementary Material S2.

### 3.3. Quality of the Included Studies

Most of the studies met at least 50% of the assessed quality criteria. The quality scores of the cross-sectional studies ranged from 45.45% to 84.5%, with an average quality rating of 71.40%. The cohort study conducted by Shi et al. (2023) [22] achieved the highest quality score, at 94.11% (Supplementary Material S3). In contrast, the crossover interventions demonstrated quality scores between 54.5% and 72.73%, with a mean score of 63.26% (Supplementary Material S4).

### 3.4. Risk of Bias

**Figure 2:**
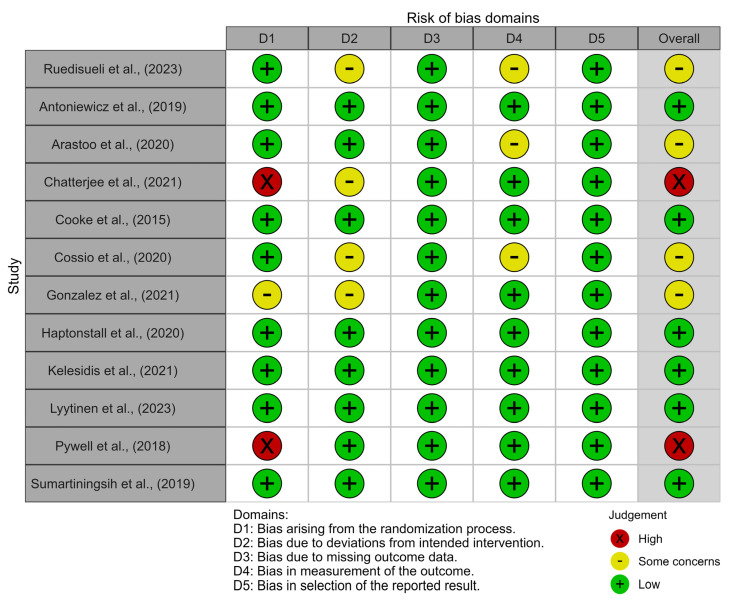
Risk of bias traffic light plot [27,29,30,31,32,33,34,35,36,37,38,40].

Only six studies were classified as having a low risk of bias [29,32,35,36,37,40], with four studies assessed as having some concerns regarding bias [27,30,33,34] and only two studies being judged to have a high risk of bias [31,38] (Figure 2).

**Figure 3:**
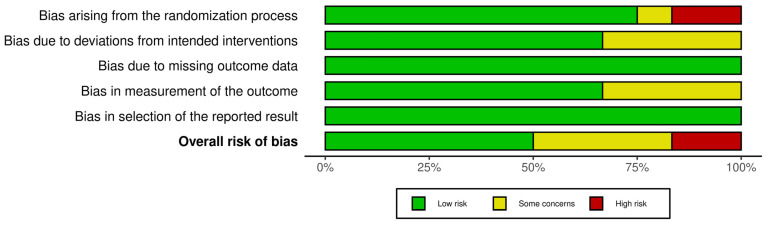
Risk of bias summary plot.

The primary source of bias was related to the randomization process, with some bias in the deviation from intervention and measurement of outcome domains. In contrast, the domains of outcome selection and data reporting exhibited the lowest risk of bias (Figure 3).

### 3.5. Narrative Synthesis of Individual Study Results

Of the included studies, 10 reported adverse effects associated with the use of ECs [20,21,23,26,27,28,29,31,32,34]. Two studies specifically attributed these adverse effects to the presence of nicotine in EC components [37,38]. Conversely, two studies found no significant differences when compared with CT use or to a control group [27,33]. Finally, seven studies indicated that EC use was associated with fewer harmful effects compared to CT [22,24,25,30,35,40].

#### 3.5.1. Dysfunction of Endothelial Vasodilatation

Three of the studies assessed endothelial function in regular EC users and reported impairments in nitric oxide-dependent vasodilatory function [20,23,34]. In contrast, the study by Haptonstall et al. (2020) [35] found no evidence of endothelial dysfunction in an acute comparison between ECs and CT. Additionally, Pywell et al. (2018) [38] concluded that the observed vasodilatory impairment was attributable to the effect of nicotine.

#### 3.5.2. Increase in Blood Pressure and Heart Rate

In the studies conducted by Cooke et al. (2015) [32] and Gonzalez and Cooke (2021) [34], exposure of healthy young non-smokers to a single session of EC inhalation resulted in elevated blood pressure and HR. In contrast, Sumartiningsih et al. (2019) [40] reported that such increases were observed with CT use, but not with ECs. Similarly, Shi et al. (2023) [22] found that elevated blood pressure was associated with CT smoking, but not with EC use or among non-smokers. Finally, the study by Cossio et al. (2020) [33] found no significant differences in blood pressure or HR between groups.

#### 3.5.3. Increase in Cellular Oxidative Stress

Moheimani et al. (2017) [26] reported that EC users exhibited higher plasma levels of biomarkers associated with cellular oxidative stress compared to non-smokers. Similarly, Kelesidis et al. (2021) [36] observed an increase in these oxidative stress markers following ENDS inhalation; however, the magnitude of this increase was lower than that observed in CT smokers.

#### 3.5.4. Increase in Systemic Inflammatory Activity

The studies by Boas et al. (2017) [24] and Chatterjee et al. (2021) [31] reported elevated levels of inflammatory and metabolic activity markers, as assessed through imaging techniques and biochemical biomarkers. These increases were more pronounced in CT smokers than in ECs users. In contrast, Ruedisueli et al. (2022) [39] found no significant differences in inflammatory activity between groups.

#### 3.5.5. Pro-Atherogenic and Pro-Thrombotic Effects

Kelesidis et al. (2023) [25] observed an increase in cells involved in atherogenesis among ENDS users, although the effect was more pronounced in CT smokers. Similarly, Lyytinen et al. (2023) [37] reported enhanced platelet activation and thrombus formation, following exposure to nicotine-containing ECs. These pro-thrombotic effects were attributed primarily to the action of nicotine and may contribute to the overall pro-atherogenic risk profile associated with ECs use.

#### 3.5.6. Prolongation of Ventricular Repolarization Time

Ruedisueli et al. (2023) [27] found, through electrocardiographic analysis, that acute ENDSs exposure in chronic CT smokers led (only in men) to a greater prolongation of ventricular repolarization times, a recognized risk factor for sudden cardiac death.

#### 3.5.7. Heart Rate Variability

Moheimani et al. (2017) [26] found a shift toward sympathetic dominance and reduced vagal tone in habitual ENDS users, a pattern linked to increase cardiovascular risk. Similarly, Sumartiningsih et al. (2019) [40] observed that acute nicotine exposure from ENDSs and CT increased HR variability parameters during exercise, indicating heightened autonomic modulation.

### 3.6. Meta-Analysis

#### 3.6.1. SBP (Nicotine vs. No Nicotine)

A meta-analysis comparing SBP responses between nicotine and non-nicotine conditions showed that, overall, nicotine exposure significantly increased SBP compared to non-nicotine ECs (MD = 3.14 mmHg; 95% CI: 5.52 to 0.76; *p* = 0.010).

**Figure 4:**
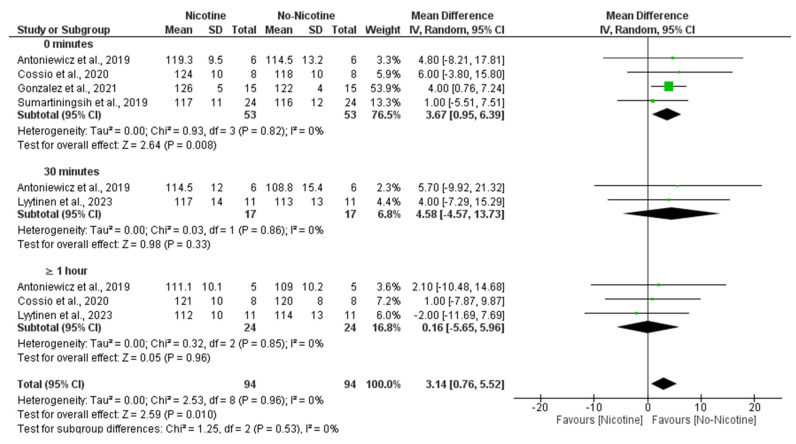
Forest plot of the effects of nicotine versus no nicotine on systolic blood pressure at different post-exposure time points [29,33,34,37,40]. Green squares show each study’s weighted mean difference, and black diamonds show the pooled mean difference with its 95% confidence interval.

This effect was most evident immediately after acute exposure (0 min), with a significant increment in the nicotine condition (MD = 3.67 mmHg; 95% CI: 0.95 to 6.39; *p* = 0.008). No significant differences were found at 30 min (MD = 4.58 mmHg; 95% CI: −4.57 to 13.73; *p* = 0.33) or ≥1 h (MD = 0.16 mmHg; 95% CI: −5.65 to 5.96; *p* = 0.96) post-exposure. There was no evidence of heterogeneity across studies (I^2^ = 0%), and subgroup analysis revealed no significant differences between time points (Figure 4).

**Figure 5:**
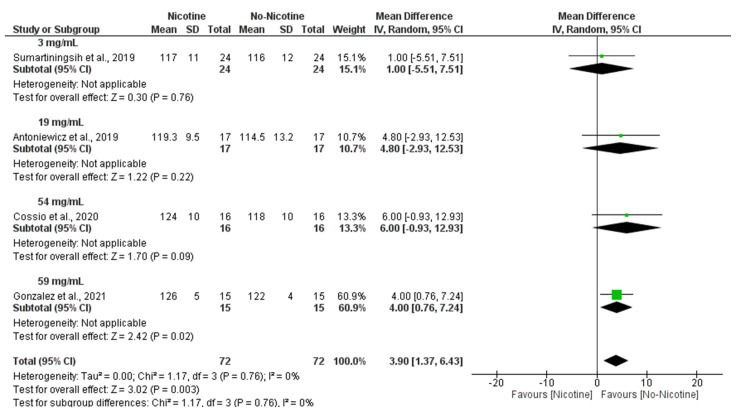
Forest plot of the acute effects of nicotine versus no nicotine on systolic blood pressure at different nicotine concentrations [29,33,34,40]. Green squares show each study’s weighted mean difference, and black diamonds show the pooled mean difference with its 95% confidence interval.

Moreover, subgroup analyses indicated that the nicotine concentration within e-cigarettes also played a significant role, with higher doses being associated with greater increases in SBP, particularly at 59 mg/mL (MD = 4.00 mmHg; 95% CI: 0.76 to 7.24), contributing to the overall significant effect (MD = 3.90 mmHg; 95% CI: 1.37 to 6.43; *p* = 0.003) with negligible heterogeneity (I^2^ = 0%) (Figure 5).

#### 3.6.2. DBP (Nicotine vs. No Nicotine)

**Figure 6:**
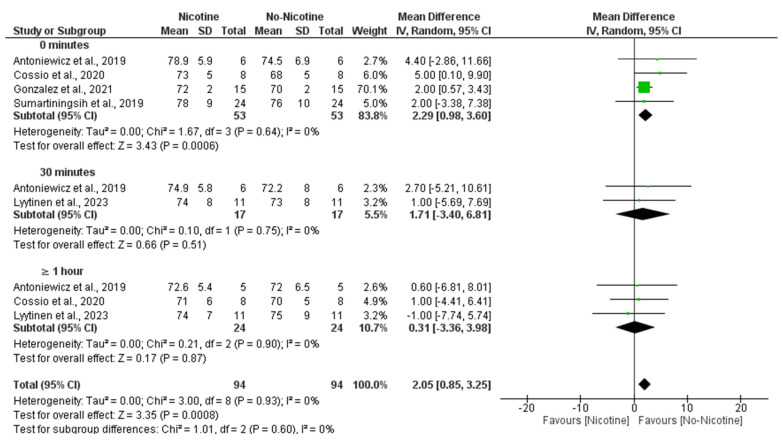
Forest plot of the effects of nicotine versus no nicotine on diastolic blood pressure at different post-exposure time points [29,33,34,37,40]. Green squares show each study’s weighted mean difference, and black diamonds show the pooled mean difference with its 95% confidence interval.

A meta-analysis examining DBP revealed that nicotine exposure significantly increased DBP compared to non-nicotine ECs (MD = 2.05 mmHg; 95% CI: 0.85 to 3.25; *p* = 0.0008). This effect was primarily observed immediately after exposure (0 min), where the nicotine group showed significantly higher DBP values (MD = 2.29 mmHg; 95% CI: 0.98 to 3.60; *p* = 0.0006). No significant differences were found at 30 min (MD = 1.71 mmHg; 95% CI: −3.40 to 6.81; *p* = 0.51) or ≥1 h post-exposure (MD = 0.31 mmHg; 95% CI: −3.36 to 3.98; *p* = 0.87). Heterogeneity was negligible across studies (I^2^ = 0%), and no significant subgroup differences were detected across time points (Figure 6).

**Figure 7:**
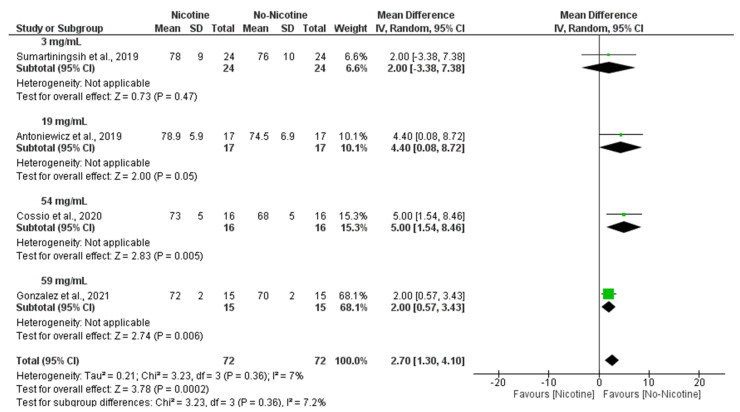
Forest plot of the acute effects of nicotine versus no nicotine on diastolic blood pressure at different nicotine concentrations [29,33,34,40]. Green squares show each study’s weighted mean difference, and black diamonds show the pooled mean difference with its 95% confidence interval.

Similarly, subgroup analyses demonstrated that nicotine concentration was a significant determinant of DBP responses, with the strongest effects observed at 54 mg/mL (MD = 5.00 mmHg; 95% CI: 1.54 to 8.46; *p* = 0.005) and 59 mg/mL (MD = 2.00 mmHg; 95% CI: 0.57 to 3.43; *p* = 0.006), leading to an overall significant increase in DBP following nicotine exposure (MD = 2.70 mmHg; 95% CI: 1.3 to 4.10; *p* = 0.0002) with very low heterogeneity (I^2^ = 7%) (Figure 7).

#### 3.6.3. Heart Rate (Nicotine vs. No Nicotine)

**Figure 8:**
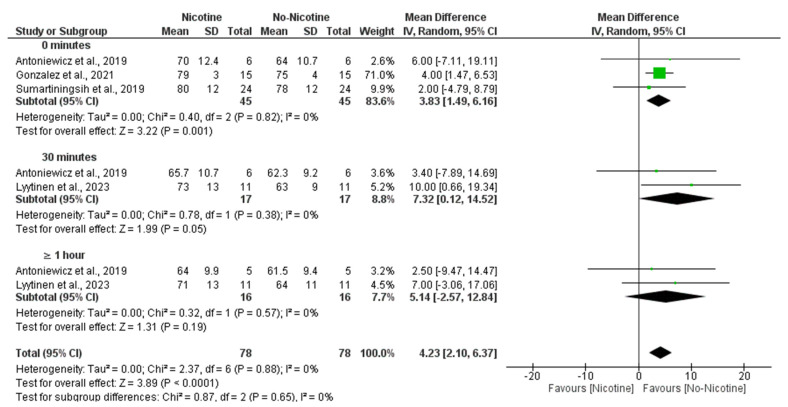
Forest plot of the effects of no nicotine versus control on heart rate at different post-exposure time points [29,34,37,40]. Green squares show each study’s weighted mean difference, and black diamonds show the pooled mean difference with its 95% confidence interval.

The meta-analysis demonstrated that nicotine exposure significantly increased HR compared to non-nicotine ECs (MD = 4.23 bpm; 95% CI: 2.10 to 6.37; *p* < 0.0001). The most prominent effect was observed immediately after exposure (0 min), with significantly higher HR in the nicotine condition (MD = 3.83 bpm; 95% CI: 1.49 to 6.16; *p* = 0.001). A significant difference was also observed at 30 min (MD = 7.32 bpm; 95% CI: 0.12 to 14.52; *p* = 0.05), although the confidence interval was wide and nearly included the null. At ≥1 h post-exposure, no significant difference was found (MD = 5.14 bpm; 95% CI: −2.57 to 12.84; *p* = 0.19). There was no heterogeneity across studies (I^2^ = 0%), and subgroup analysis showed no statistically significant differences between time points (Figure 8).

**Figure 9:**
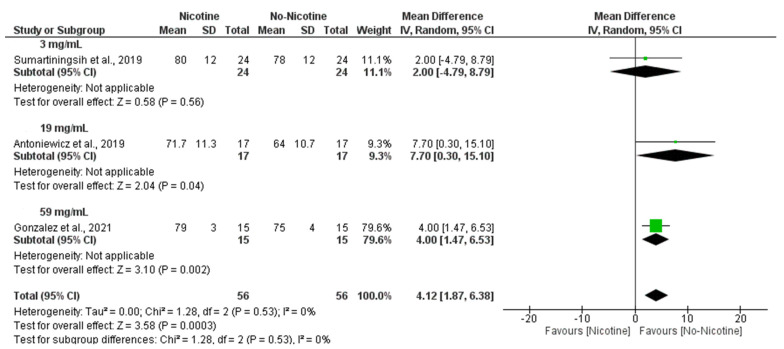
Forest plot of the acute effects of nicotine versus no nicotine on heart rate at different nicotine concentrations [29,34,40]. Green squares show each study’s weighted mean difference, and black diamonds show the pooled mean difference with its 95% confidence interval.

In line with these findings, subgroup analyses revealed that nicotine concentration also influenced HR responses, with 59 mg/mL nicotine conditions producing a significant increase compared to non-nicotine conditions (MD = 4.00 bpm; 95% CI: 1.47 to 6.53; *p* = 0.002), while 19 mg/mL nicotine conditions showed the larger MD, with its confidence interval nearly including the null hypothesis (MD = 7.70 bpm; 95% CI: 0.30 to 15.10; *p* = 0.04). Altogether, this resulted in an overall significant increase in HR in the nicotine group compared with the non-nicotine group (MD = 4.12 bpm; 95% CI: 1.87 to 6.38; *p* < 0.001) with no heterogeneity (I^2^ = 0%) (Figure 9).

## 4. Discussion

The main objective of this systematic review and meta-analysis was to compile the most recent evidence on the cardiovascular effects of EC use in young populations. The findings indicate that, although most of the included studies reported cardiovascular effects associated with EC use, some discrepancies were observed across studies. Overall, cardiovascular effects were more consistently observed and of greater magnitude with CT consumption compared to ECs.

Additionally, the study aimed to assess whether the effects resulting from EC inhalation were linked to acute use, chronic use, or both and the time- and dose-dependent cardiovascular effects. The findings derived from the reviewed literature indicate that acute exposure to these devices was associated with the following cardiovascular effects: activation of the sympathetic nervous system and reduced vagal tone [26], heightened autonomic modulation during physical exercise [40], impaired endothelium-dependent vasodilatation mediated by nitric oxide [20,23,28], increased blood pressure and HR [26,29,30,32,40], elevated cellular oxidative stress [26], enhanced inflammatory activity [21,31] and increased thrombotic and platelet activity at the microcirculatory level [37]. In the narrative synthesis, some discrepancies were noted regarding the influence of nicotine on the increases in blood pressure and HR. However, the meta-analysis indicated that these cardiovascular changes were primarily attributed to the nicotine content in these ENDSs. Most of these acute effects are consistent with those reported by Siddiqi et al. (2023) [12] in their 2023 meta-analysis conducted in populations of varying ages.

Regarding chronic exposure to ENDS vaping, the most consistently reported findings associated with months-long use of ECs included a reduction in endothelium-dependent vasodilatation mediated by nitric oxide [20,23,28], and the presence of atherosclerotic plaque formation [36]. It could be hypothesized that acute increase in BP, if sustained over time, may contribute to the development of hypertension in the future. Additionally, a prolongation of ventricular repolarization has been observed in EC vapers [27]. However, despite the documented increases in inflammatory, thrombotic, and/or platelet activity, as well as the prolongation of ventricular repolarization, which is known to promote arrhythmias, this systematic review did not identify any scientific literature reporting clusters of serious and/or fatal cardiovascular events in adolescents or young adults who use ECs. This stands in contrast to the pulmonary damage observed in the so-called EVALI syndrome, first reported by the US Centers for Disease Control and Prevention (CDC) in 2019 [41]. In this regard, Adkins et al., in their 2019 study, included hospitalized or deceased adolescents and concluded that the use of any EC or vaping product is unsafe [42].

The rationale for investigating acute exposure lies in the increasing trend of “weekend” or occasional use of e-cigarettes during social events among many contemporary European and North American youth. A striking finding from the meta-analysis was the time- and dose-dependent effects of ENDSs on the increases in blood pressure (both systolic and diastolic) and HR after vaping, compared to nicotine-free devices. These elevations tend to normalize within 30 to 60 min post-inhalation. While this transient effect might appear to trivialize ENDS use or even promote it, it is crucial to emphasize that smoking behaviour also involves elements of “orality” and “gesturally” that can reinforce or introduce active CT smoking habits. Consequently, dual use alongside CT, or even initiation into CT use, cannot be ruled out, particularly in young individuals who, due to their age, represent long-term target population for the tobacco industry. In this regard, even Hajek et al. (2019) concluded that ECs may hold greater potential for leading to regular use among non-smokers [5]. The seemingly lower harm profile of e-cigarettes compared to combustible tobacco is one of the key arguments used by proponents who advocate for their use as a smoking cessation aid, a practice permitted in such countries as the United Kingdom, Norway, and Belgium [43]. In relation to this, Pearson et al. (2020), in a cohort study conducted among American youth, found that EC use was neither associated with an increase nor a reduction in CT consumption after one year [44]. This is perhaps because most adolescents and young smokers are in the pre-contemplative stage of change phase [45]. In contrast, EC use is prohibited by law in some countries, like Spain, where these devices are not considered medical products [46]. In this context, legislation is increasingly being developed to regulate ECs with the aim of ensuring their safe use [43] or even prohibiting their use.

A third objective of this study was to compare the cardiovascular damage associated with EC use to that caused by CT consumption, in order to determine whether the impact on the cardiovascular system was greater than, lesser than, or comparable to that of CT. Most studies comparing both types of cigarettes reported greater cardiovascular damage associated with CT use [22,24,25,30,35,36,40] while only three studies associated greater damage with EC use [21,27,28]. Ruediseli et al. (2022) did not observe differences in metabolic activity, underscoring the role of the sympathetic nervous system in provoking inflammatory monocyte proliferation, thus instigating atherosclerotic development [39].

Notably, the study of Shi et al. stands out for two reasons. First, it was the only cohort study to analyze the effects retrospectively. Secondly, it had a large sample size and high-quality score. It is noteworthy that in this study, neither EC use nor dual use were associated with an increased risk of hypertension in either men or women, but CT use was associated with a higher risk of hypertension in female smokers, as a relevant result from a gender perspective. The hypertension effect was also seen in EC users [22], although this information on clinically relevant adverse effects of EC users is weak, mostly because of a lack of powerful studies. Further exploring this gender perspective, Halstead et al. reported endothelial-dependent VD dysfunction due to an excess of reactive oxygen species in plasma, which was more pronounced in women compared to men [23]. Conversely, the prolongation of ventricular repolarization indices, highlighted in the study by Ruedisueli et al. (2023) [27], was observed only in men vaping with ECs.

Regarding the non-nicotine components of ECs, the study by Chatterjee S et al. focused on assessing the effects of acute inhalation of nicotine-free EC aerosol, with the aim of determining whether other EC constituents exert toxic effects [31]. This study found an increase in inflammatory markers, that impaired endothelial response to increased flow reduced hyperemic response following vascular occlusion, and additional metabolic disturbances, including reduced venous oxygen saturation. The damage caused by these non-nicotine components, present in EC liquids, has been analyzed in previous studies, which demonstrated their toxicity through the exposure of cultured cells to EC liquid in vitro [47,48,49]. Among these components, propylene glycol and glycerol stand out, as they can induce apoptosis of the cells they contact and cause other adverse effects, such as mitochondrial dysfunction, particularly when combined with other compounds in flavored EC liquids, such as certain aldehydes [47,48,49]. Nonetheless, it is important to consider users’ health habits and to clarify whether the observed adverse effects are attributable solely to nicotine or also to other excipients, or even to the contact of the generated aerosol with the body.

Despite the rigorous methodological approach, this meta-analysis has several limitations. The number of studies included in certain comparisons was limited, potentially reducing the statistical power and the precision of the pooled estimates, and precluding the exploration of heterogeneity through meta-regression. This may be because additional databases, such as EMBASE or CINHAL, as well as the gray literature, were not searched. Moreover, individual studies often relied on small, non-representative samples, with minimal stratification by sex, ethnicity, or other relevant demographic variables. Among the main ones is the heterogeneity observed in the comparison groups across the included studies. Not all selected studies compared young CT smokers, EC users, and non-smokers simultaneously, which may affect the interpretation of the findings. Additionally, while some studies focused exclusively on healthy non-smokers acutely exposed to ECs, others evaluated chronic users. There was also significant variability in the cardiovascular outcomes and measurements techniques used across studies. Future studies employing more standardized and comparable variables are needed to enable more precise conclusions regarding the cardiovascular effects of EC use in young adults. Moreover, this review did not specifically examine gender-related differences, which may influence the observed effects and limit the generalizability of the findings; future meta-analysis should assess sex differences in EC use and cardiovascular related effects.

## 5. Conclusions

The evidence synthesized in this systematic review and meta-analysis suggests that EC use among young individuals is associated with modest cardiovascular effects, which appear more consistently following acute exposure. These effects, including transient increases in BP and HR, were more pronounced in nicotine-containing ECs as the nicotine dose increased. However, the absolute effect sizes were generally small.

Importantly, the available literature is largely based on acute and cross-sectional data, with a scarcity of robust longitudinal studies evaluating long-term cardiovascular outcomes in young populations. Therefore, the observed associations should not be interpreted as causal.

Based on the current evidence, both ECs and CTs should be considered unsafe from a cardiovascular health perspective, and their use should be approached with caution, especially in young populations. These findings may contribute to the development of future public health policies and preventive strategies.

## Figures and Tables

**Table 1:** Characteristics of the included studies.

Author, Year	Study Type	Groups	Sample Size	Mean Age	Sex (F/M)	EC Nicotine (mg/mL) *	Measured Effects	Assessed Variable	Results
Antoniewicz L et al. (2019) [29]	Randomized, double-blinded, crossover study	Non- smokers	15	26 ± 3	9/6	19	Vascular and pulmonary function following exposure to EC inhalation with or without nicotine	PWV (m/s)	Inhalation of ECs with and without nicotine led to an increase in BP. However, HR and arterial stiffness increased only with nicotine-containing ECs.
								SBP (mmHg)	
								DBP (mmHg)	
								HR (bpm)	
Arastoo S et al. (2020) [30]	Open-label randomized crossover study	EC	58	28 ± 5	19/39	50	Sympathetic activation following exposure to inhalation sessions with nicotine-containing ECs, ECs without nicotine, nicotine inhalers, and CTs	SBP (mmHg)	EC and CT users experienced increases in BP and HR following inhalation of ECs and CT, respectively. These changes were more pronounced among participants who inhaled CT and were primarily attributed to the effects of nicotine.
		CT	42	27 ± 6	15/27			DBP (mmHg)	
								HR (bpm)	
Boas et al. (2017) [24]	Cross-sectional study based on an experimental protocol	EC	9	29 ± 1.5	2/7	NE	Metabolic activity of the splenocardiac axis assessed by PET-FDG	SUVmax	Both CT and EC showed increased FDG uptake in the spleen and aorta, with a greater increase observed in CT smokers.
		CT	9	27 ± 1.5	2/7			Paraoxonase 1 enzyme activity (nmol p-nitrophenol/min/mL)	
		Non- smokers	9	28 ± 1.5	3/6				
Chatterjee S et al. (2021) [31]	Experimental repeated-measures study	Non- smokers	31	24 ± 4	14/17	0	Changes in inflammation and oxidative stress assessed by blood biomarkers and MRI following acute inhalation of ECs	CRP (ng/mL)	Subjects exposed to ECs exhibited increases in CRP, ICAM, ASC, and HMGB1 levels, along with a decrease in venous oxygen saturation and alterations in vascular diameter and resistance.
								ICAM (ng/mL)	
								HMGB1 (ng/mL)	
								ASC containing a CARD (ng/mL)	
								NO (μmol/L)	
								FMD (%)	
								PWV (m/s)	
								Pulsatility index (dimensionless)	
Cooke WH et al. (2015) [32]	Randomized, counterbalanced, and double-blinded pilot study	Non- smokers	20	23 ± 1	10/10	18	Changes in BP, HR, and autonomic cardiovascular control following EC inhalation	HR (ECG R–R interval)	Acute nicotine inhalation increased SBP and DBP, as well as HR, but did not affect autonomic cardiovascular control under conditions of orthostatic stress.
								Autonomic nervous system modulation (bursts/min)	
								SBP (mmHg)	
								DBP (mmHg)	
Cossio R et al. (2020) [33]	Randomized crossover study	Non- smokers	16	24 ± 3	7/9	54	Vascular function: HR and BP following inhalation sessions with nicotine-containing and nicotine-free ECs	FMD (%)	EC with and without nicotine did not produce any significant changes in HR, SBP, DBP, or endothelial function.
								SBP (mmHg)	
								DBP (mmHg)	
Gonzalez JE et al. (2021) [34]	Randomized crossover study	Non- smokers	15	21 ± 1	6/9	59	Changes in BP and ANSM following exposure to a single inhalation session of EC with and without nicotine	SBP (mmHg)	Inhalation of nicotine-containing EC increased BP and HR, along with a reduction in ANSM modulation. Inhalation of the placebo EC had no effect on sympathetic activity.
								DBP (mmHg)	
								HR (bpm)	
								ANSM (bursts/min)	
								Cardiovagal reflex sensitivity (ms/mmHg)	
Halstead KM et al. (2023) [23]	Cross-sectional study	EC	20	21 ± 2	10/10	NE	Endothelium-dependent VD assessed by measuring CVC responses to local heating with tempol	CVC (%)	EC users exhibited a reduction in NO-dependent VD compared to non-smokers, attributed to an excess of reactive oxygen species in plasma. Differences were more pronounced in women.
		Non- smokers	20	21 ± 2	10/10			Percentage of NO-dependent vasodilatation (%)	
Haptonstall KP et al. (2020) [35]	Open-label, randomized crossover study	EC	49	27 ± 5	13/36	50	Endothelial VD function following exposure to EC, CT, and a placebo	FMD (%)	EC use did not alter FMD, unlike CT inhalation, which induced endothelial dysfunction, consistent with the notion that non-nicotine constituents in CT smoke mediate the impairment.
								HR (bpm)	
								SBP (mmHg)	
		CT	40	27 ± 5	14/26			DBP (mmHg)	
								MAP (mmHg)	
		Non- smokers	47	26 ± 5	25/22			Shear stress (velocity/diameter)	
								SSIRH (dynes/cm^2^)	
								Reactive hyperemia velocity (cm/s)	
Kelesidis T et al. (2021) [36]	Randomized clinical crossover trial	EC	12	24 ± 4	13/19	50	Changes in oxidative stress cell counts in blood following a single inhalation session of EC	Counts of blood CD45, CD16, CD14 monocytes, T cells, and natural killer (NK) cells	Both EC and CT users exhibited increased levels of oxidative stress compared to non-smokers. However, CT users showed a greater increase than EC users.
		CT	9	25 ± 4					
		Non- smokers	11	24 ± 2					
Kelesidis T et al. (2023) [25]	Cross-sectional study	EC	21	23 ± 1	12/9	NE	Pro-atherogenic events assessed by plasma measurement of parameters related to transendothelial monocyte migration and foam cell formation	Percentage of transendothelial migration of macrophages and monocytes	Transendothelial migration of monocytes and macrophages, as well as foam cell formation, were significantly increased in EC users compared to non-smokers. However, these increases were significantly lower than those observed in CT users.
		CT	18	24 ± 1	9/9				
		Non- smokers	21	23 ± 2	10/11				
Lyytinen G et al. (2023) [37]	Randomized double-blind crossover study	Occasional smokers or snus users	22	27 ± 7	15/7	19	Fibrin and platelet thrombus formation following exposure to EC with and without nicotine, with subsequent measurement of plasma markers	Time to reach 10 kPa (s)	Inhalation of nicotine-containing EC, compared to nicotine-free EC, resulted in increased platelet and fibrin thrombus formation, and a reduction in microcirculatory VD capacity.
								Time to reach occlusion pressure (s)	
								Baseline blood flow and after 30 min infusion of acetylcholine or SNP	
Matheson C et al. (2024) [20]	Cross-sectional study	EC	21	23 ± 3	11/10	NE	Vascular function of subjects assessed by vascular reactivity tests	Red blood cell flow (UP)	EC users exhibited lower levels of hyperemia following vascular occlusion, reduced VD in response to acetylcholine infusion, and decreased heat-induced VD compared to controls. No differences were observed in endothelium-independent VD.
								CVC (UP/mmHg)	
		Non- smokers	21	25 ± 5	12/9			FMD (%)	
Moheimani R et al. (2017) [26]	Cross-sectional case–control study	EC	16	29 ± 1	3/13	NE	Variations in HR and oxidative stress assessed through plasma parameters	HR (bpm)	EC use was associated with an increase in HR and elevated oxidative stress levels compared to the non-smoking group Habitual EC use was associated with a shift in cardiac autonomic balance toward sympathetic predominance and increased oxidative stress, both associated with increased cardiovascular risk.
								LDL oxidative capacity (dimensionless)	
								HDL antioxidant index (dimensionless)	
		Non- smokers	18	27 ± 1	11/7			Paraoxonase-1 activity (nmol p-nitrophenol/min/mL)	
								Plasma fibrinogen (mg/dL)	
Pywell MJ et al. (2018) [38]	Pilot experimental repeated-measures study	CT	7	26 ± 1	NE	24	Hand microvascular blood flow assessed by Doppler following inhalation of EC with and without nicotine	Superficial and deep blood flow (arbitrary units, equivalent to ml/min)	CT smokers exhibited a decrease in hand microvascular blood flow during inhalation of nicotine-containing ECs.
		Non- smokers	8	25 ± 3					
Ruedisueli I et al. (2023) [27]	Randomized crossover experimental study with a cross-sectional baseline comparison	EC	34	23 ± 3	17/17	50	Changes in ventricular repolarization assessed by ECG in subjects following inhalation of CT and EC	Tp-e interval (ms)	Inhalation of ECs, unlike CT inhalation, resulted in prolongation of ventricular repolarization indices. This effect was observed only in men and not in women.
								Tp-e/QT ratio	
		CT	35	24 ± 3	21/14			Tp-e/QTc ratio	
								QT (ms)	
		Non- smokers	41	24 ± 4	21/20			QTc (ms)	
								HR (bpm)	
Ruedisueli S et al. (2022) [39]	Cross-sectional comparative	EC	16	25 ± 4	6/10	NE	Metabolic activity of the splenocardiac axis, associated with sympathetic activity and atherosclerosis development, assessed by PET-FDG	SUVmax	Underscored the role of the sympathetic nervous system in provoking inflammatory monocyte proliferation, instigating atherosclerotic development. No differences were observed in metabolic activity among the subjects studied.
		CT	14	27 ± 6	7/7				
		Non- smokers	15	25 ± 4	6/9				
Sahota A et al. (2021) [21]	Cross sectional study	EC	20	26 ± 4	7/13	NE	Presence of atherosclerosis parameters in the carotid artery assessed by PET-MRI	SUVmax	CT and EC smokers exhibited atherosclerotic plaques in carotid MRI images, unlike the non-smoker group. This damage was more pronounced in EC smokers.
		CT	20	27 ± 3	7/13				
		Non- smokers	20	25 ± 2	8/12				
Shi H et al. (2023) [22]	Prospective longitudinal cohort study	EC	372	18 to 24	177/196	NE	Development of HTN following exclusive use of EC, CT, or dual consumption of both, based on a retrospective analysis of survey data	HTN yes/no	Exclusive use of CT was associated with an increased risk of HTN in women. Neither EC use nor combined EC and CT consumption were associated with HTN risk in either men or women.
		CT	4933		2573/2360				
		EC + CT	573		291/283				
		Non- smokers	10486		5725/ 4761				
Sumartiningsih S et al. (2019) [40]	Randomized crossover experimental study with repeated measures	EC CT	24	23 ± 2	0/24	3	Cardiac response to exercise following exposure to nicotine-free EC, nicotine-containing EC, and CT	HR (bpm)	Inhalation of CT resulted in an increase in HR and a decrease in DBP. Significant acute autonomic cardiac modulation during exercise was induced by acute episodes of EC and CT smoking.
								SBP (mmHg)	
								DBP (mmHg)	
								Maximal oxygen consumption (ml/kg/min)	
								Time to exhaustion (sec)	
Youn JY et al. (2023) [28]	Cross sectional study	EC	13	22 ± 1	6/7	NE	Endothelial damage function assessed through plasma nitrite levels	Circulating plasma nitrite levels (µM)	Nitrite levels were lower in EC users compared to both the control and CT groups.
		CT	11	23 ± 1	6/5				
		Non- smokers	9	22 ± 1	3/6				

ASC = apoptosis-associated speck-like protein; ANSM = autonomic nerve system modulation; BP = blood pressure; BPM = beats per minute; cm = centimeters; CRP = C-reactive protein; CT = combustion tobacco; CVC = cutaneous vascular conductance; DBP = diastolic blood pressure; EC = electronic cigarette; ECG = electrocardiogram; FDG = fluorodeoxyglucose; FMD = flow-mediated dilation; HMGB1 = high mobility group box 1 protein; HR = heart rate; HTN = hypertension; Hz = hertz; ICAM = intercellular adhesion molecule; kg = kilograms; kPa = kilopascals; m = meters; min = minute; ml = milliliters; mmHg = millimeters of mercury; MRI = magnetic resonance imaging; NE = not specified; ng = nanograms; nmol = nanomoles; NO = nitric oxide; PET = positron emission tomography; PWV = pulse wave velocity; QT = electrocardiogram QT interval; QTc = corrected QT interval; SBP = systolic blood pressure; SSIRH = shear stress-induced reactive hyperemia; SNP = sodium nitropusside; sec = second; SUV = standardized uptake value; SUVmax = maximum standardized uptake value; Tp-e = interval from T-wave peak to end; PU = perfusion unit; VD = vasodilation; µM = micromolar; µs^2^ = microseconds squared. * = although mg/mL of nicotine was the initial unit, some studies used other units of measurement (e.g., cotinine, JUUL).

## Data Availability

No new data were created or analyzed in this study. Data sharing is not applicable to this article.

## Supplementary Materials

The following supporting information can be downloaded at: https://www.mdpi.com/article/10.3390/toxics13100831/s1, Supplementary material S1: Search Engine; Supplementary material S2: Specific Design Characteristics; Supplementary material S3: Strobe Scale; Supplementary material S4: CONSORT scale.

## Abbreviations

The following abbreviations are used in this manuscript:

µM	Micromolar
ANSM	Autonomic nervous system modulation
ASC	Apoptosis-associated speck-like protein containing a CARD
bpm	Beats per minute
CRP	C-reactive protein
CT	Conventional tobacco
CVC	Cutaneous vascular conductance
DBP	Diastolic blood pressure
EC	Electronic cigarette
ENDS	Electronic nicotine delivery system
EVALI	E-cigarette or vaping-associated lung injury
FMD	Flow-mediated dilation
HMGB1	High-mobility Group Box 1 Protein
HR	Heart rate
ICAM	Intercellular adhesion molecule
MAP	Mean arterial pressure
mmHg	Millimeters of mercury
MRI	Magnetic resonance imaging
ng/mL	Nanograms per milliliter
nmol	Nanomole
NO	Nitric oxide
PET	Positron emission tomography
PU	Perfusion unit
PWV	Pulse wave velocity
QT	Electrocardiogram QT interval
QTc	Corrected QT interval
RCT	Randomized controlled trial
SBP	Systolic blood pressure
SUVmax	Maximum standardized uptake value
Tp-e	T-wave peak to end interval
