# Supplementary material for: Time- and Dose-Dependent Cardiovascular Effects of Nicotine-Containing Electronic Cigarettes in Young Adults: A Systematic Review and Meta-Analysis
Source: Toxics. 2025 Sep 30;13(10):831. doi: 10.3390/toxics13100831 (PMC12567738; doi:10.3390/toxics13100831)
Supplement: Supplementary file 1 [file toxics-13-00831-s001.zip › Supplementary Materials S1 Search Engine.pdf]

Supplementary Materials S1: Search Engine

**Pubmed**	*((((electronic nicotine delivery systems) AND (young adults)) AND (effects OR injury OR health)) NOT (perceptions)) NOT (prevalence)*
**Scopus**	*(ALL ("electronic nicotine delivery systems" AND "young adults") AND ALL ("effects" OR "injury" OR "health") AND NOT ALL ("perceptions" OR "prevalence"))*
**Web of Science**	*((((ALL= ("electronic nicotine delivery systems")) AND ALL= ("young adults")) AND ALL= (effects OR injury OR health)) NOT ALL=(perceptions)) NOT ALL=(prevalence)*
