# Supplementary material for: Time- and Dose-Dependent Cardiovascular Effects of Nicotine-Containing Electronic Cigarettes in Young Adults: A Systematic Review and Meta-Analysis
Source: Toxics. 2025 Sep 30;13(10):831. doi: 10.3390/toxics13100831 (PMC12567738; doi:10.3390/toxics13100831)
Supplement: Supplementary file 1 [file toxics-13-00831-s001.zip › Supplementary Materials S2 Specific design characteristics.pdf]

Supplementary Materials S2: Specific Design Characteristics

**Author, year**	**Study design characteristics**
Antoniewicz L et al., (2019) [28]	Vascular outcomes (heart rate, systolic and diastolic blood pressure, and arterial stiffness) and pulmonary outcomes (dynamic spirometry, impulse oscillometry, and fractional exhaled nitric oxide) were assessed. Vascular measurements were taken at baseline and subsequently every 10 minutes for 30 minutes after the inhalations, which were performed at 0 h (immediately post-exposure), 2 h, and 4 h. Participants were randomized to two conditions (electronic cigarette with nicotine and electronic cigarette without nicotine) and, after a one-week washout, repeated the experiment in the opposite group.
Arastoo S et al., (2020) [29]	This open-label randomized crossover study included chronic EC users who underwent up to four 30-min acute exposure sessions in random order, separated by 4 weeks: sham vaping (empty EC), EC with nicotine, EC without nicotine, and nicotine inhaler (a clean nicotine source without flavorings or solvents).
Boas et al., (2017) [23]	A total of 31 participants were enrolled (10 habitual tobacco cigarette users, 11 habitual e-cigarette users, and 10 healthy controls); nine from each group were included in the final analysis. Measurements were performed after 90 min of rest, with high-count 5-min scans per bed position of the neck, chest, and abdomen, providing improved image quality for reliable and reproducible quantitative assessment.
Chatterjee S et al., (2021) [30]	Healthy nonsmokers underwent an e-cigarette challenge with a nicotine-free device. Each participant had two blood draws (pre- and 1–1.5 h post-vaping) and two MRI scans (before and after the challenge). The vaping session lasted 3–5 min, with the sequence consisting of blood draw → MRI → vaping challenge → MRI → blood draw.
Cooke WH et al., (2015) [31]	Participants completed two experimental sessions in randomized order, inhaling from an e-cigarette cartridge with nicotine (18 mg) or a placebo cartridge without nicotine (0 mg).
Cossio R et al., (2020) [32]	Participants completed three randomized interventions (control menthol-flavored cigarette-like pipe, nicotine EC, and non-nicotine EC) following a standardized 6-min vaping protocol (4-s inhalations every 20 s; 18 puffs total). Assessments were performed immediately, 1 h, and 2 h post-exposure to evaluate vascular effects. Sessions were separated by at least 48 h, and all measurements were conducted by a single-blinded researcher.
Gonzalez JE et al., (2021) [33]	Each participant completed two randomized sessions, one with a JUUL e-cigarette containing 59 mg/mL nicotine and one with 0 mg/mL nicotine, separated by 1 month. After a 10-min baseline, participants followed a 10-min inhalation protocol (1

	inhalation every 30 s; 20 total), guided by an investigator to control breathing frequency. A 10-min recovery followed, during which symptoms were recorded.
Halstead KM et al., (2023) [22]	The study included 20 participants in the electronic cigarette (EC) group and 20 healthy control (HC) participants who were measured for every outcome.
Haptonstall KP et al., (2020) [34]	EC vapers and nonsmokers completed up to four 30-min acute exposure sessions in randomized order, separated by 4 weeks: 1) sham vaping (empty EC), 2) EC with nicotine (ECN), 3) EC without nicotine (EC0), and 4) nicotine inhaler (NI) with inactive menthol flavoring and no solvents.
Kelesidis T et al., (2021) [35]	Immune cells were collected from participants before and 4 hours after supervised sessions of either vaping with a 5% nicotine e-cigarette or sham vaping (puffing on a straw), conducted in randomized order.
Kelesidis T et al., (2023) [24]	Healthy participants were enrolled into three groups based on smoking status: 1) tobacco cigarette (TCIG) smokers with >1 year exclusive use, 2) e-cigarette (ECIG) users with >1 year exclusive use, and 3) nonsmokers with no TCIG or ECIG use >1 year.
Lyytinen G et al., (2023) [36]	This randomized crossover study blinded participants to the intervention. Participants inhaled one puff per minute from an EC (with or without nicotine) for 30 minutes, followed by post-exposure measurements. After a one-week washout, the procedure was repeated with the opposite EC condition.
Matheson C et al., (2024) [19]	Twenty-one regular EC users were compared in a single assessment with 21 demographically matched nonsmokers (age, sex, and body mass) serving as controls.
Moheimani R et al., (2017) [25]	A total of 42 participants were enrolled, including 23 habitual e-cigarette users and 19 control participants with no tobacco or e-cigarette use. After abstaining from caffeine and e-cigarettes for ≥12 hours, participants were placed supine in a quiet, temperature-controlled (21°C) room for measurements.
Pywell MJ et al., (2018) [37]	Fifteen healthy participants (median age 26 years; 7 smokers, 8 nonsmokers) completed two 5-min smoking sessions with e-cigarettes: non-nicotine (0 mg) and nicotine (24 mg). Continuous microcirculation measurements were taken during and for 20 min after each session, averaged over 5-min periods, and expressed as a percentage of baseline.

Ruedisueli I et a., (2023) [26]	At baseline, a cross-sectional comparison was conducted between tobacco cigarette smokers, exclusive e-cigarette users, and nonusers. Additionally, randomized crossover protocols were applied within the smoker and e-cigarette cohorts to assess acute effects of different product exposures.
Ruedisueli S et al., (2022) [38]	A cross-sectional comparative observational study including 45 healthy young adults divided into three groups: nonsmokers, electronic cigarette users, and tobacco smokers.
Sahota A et al., (2021) [20]	Young adults aged 18–30 years were divided into three age-matched groups: exclusive e-cigarette users, traditional cigarette smokers, and nonsmoking controls.
Shi H et al., (2023) [21]	Data from the nationally representative U.S. Population Assessment of Tobacco and Health (PATH) cohort were analyzed to examine associations between electronic cigarette use, cigarette smoking, dual use, and subsequent incidence of hypertension.
Sumartiningsih S et al., (2019) [39]	Each participant completed three test sessions in randomized order, separated by at least three days: 1) electronic cigarette with 0 mg nicotine (control), 2) electronic cigarette with 3 mg nicotine, and 3) tobacco cigarette delivering ~3 mg nicotine.
Youn JY et al., (2023) [27]	Measurements were performed in three demographically similar groups: conventional tobacco (CT) smokers, electronic cigarette (EC) smokers, and nonsmokers.
