# Supplementary material for: Time- and Dose-Dependent Cardiovascular Effects of Nicotine-Containing Electronic Cigarettes in Young Adults: A Systematic Review and Meta-Analysis
Source: Toxics. 2025 Sep 30;13(10):831. doi: 10.3390/toxics13100831 (PMC12567738; doi:10.3390/toxics13100831)
Supplement: Supplementary file 1 [file toxics-13-00831-s001.zip › Supplementary Materials S3 Strobe Scale.pdf]

Supplementary Materials S3: STROBE Scale for Observational Studies

	Studies								
**Items**	**Matheson C et al. (2024)**	**Youn JY et al. (2023)**	**Halstead KM et al. (2023)**	**Kelesidis T et al. (2023)**	**Sahota A et al. (2021)**	**Ruedisueli I et al. (2022)**	**Shi H et al. (2023)**	**Moheimani RS et al. (2024)**	**Boas Z et al. (2023)**
	×	×	×	✓	×	×	×	✓	×
**1b**	✓	✓	✓	×	✓	✓	✓	✓	✓
**2**	✓	✓	✓	✓	✓	✓	✓	✓	✓
**3**	✓	×	✓	✓	✓	✓	✓	✓	✓
**4**	✓	×	✓	✓	✓	✓	✓	✓	✓
**5**	✓	×	✓	×	×	✓	✓	✓	×
**6a**	✓	×	×	✓	✓	✓	✓	✓	✓
**6b**	✓	✓	✓	✓	✓	✓	✓	✓	✓
**7**	✓	×	✓	✓	✓	✓	✓	✓	✓
**8**	✓	✓	✓	✓	✓	✓	✓	✓	✓
**9**	×	×	×	×	×	×	✓	×	×
**10**	✓	×	✓	×	×	✓	×	×	✓
**11**	✓	✓	✓	×	✓	✓	✓	✓	✓
**12a**	×	✓	✓	✓	✓	✓	✓	✓	×
**12b**	✓	✓	✓	✓	✓	✓	✓	✓	✓
**12c**	NI	NI	NI	NI	NI	NI	✓	✓	NI
**12d**	✓	×	×	×	×	×	✓	✓	✓
**12e**	✓	×	×	×	×	✓	✓	✓	×
**13a**	✓	×	×	×	×	×	✓	✓	✓
**13b**	✓	×	×	×	×	×	✓	✓	✓
**13c**	×	×	×	×	×	×	✓	×	✓
**14a**	✓	✓	✓	✓	✓	✓	✓	✓	✓
**14b**	✓	NI	NI	NI	NI	NI	✓	✓	✓
**14c**	NA	NA	NA	NA	NA	NA	✓	NA	NA
**15**	✓	✓	✓	✓	✓	✓	✓	✓	✓
**16a**	✓	✓	✓	✓	✓	✓	✓	✓	✓
**16b**	✓	✓	✓	✓	✓	✓	✓	✓	✓
**16c**	NI	NI	NI	NI	NI	NI	✓	NI	NI
**17**	✓	✓	✓	✓	✓	✓	✓	✓	✓
**18**	✓	✓	✓	✓	✓	✓	✓	✓	✓
**19**	✓	×	✓	×	✓	✓	✓	✓	✓
**20**	✓	✓	✓	✓	✓	✓	✓	✓	✓
**21**	✓	×	✓	×	×	✓	✓	✓	✓
**22**	✓	✓	✓	✓	✓	✓	✓	✓	✓
**Quality Index**	27/33 (81,82%)	15/33 (45,45%)	22/33 (66,7%)	18/33 (54,54%)	20/33 (60,6%)	24/33 (72,72%)	32/34 (94,11%)	28/33 (84,85%)	27/33 (81,82%)

✓ = meets the item. × = does not meet the item. NA = not applicable due to the type of study. NI = no information provided. In studies with a case-control design rather than a cohort design, item 14c, specific to cohort studies, was not considered; therefore, these studies are evaluated based on 33 instead of 34 items.
