# Supplementary material for: Time- and Dose-Dependent Cardiovascular Effects of Nicotine-Containing Electronic Cigarettes in Young Adults: A Systematic Review and Meta-Analysis
Source: Toxics. 2025 Sep 30;13(10):831. doi: 10.3390/toxics13100831 (PMC12567738; doi:10.3390/toxics13100831)
Supplement: Supplementary file 1 [file toxics-13-00831-s001.zip › Supplementary Materials S4 CONSORT Scale.pdf]

Supplementary Materials S4: Consort Scale for clinical trials

Items	Studies											
	**Kelesidis T et al. (2021)**	**Pywell MJ et al. (2018)**	**Cossio R et al. (2020)**	**Sumartiningsih S et al. (2019)**	**Chatterjee S et al. (2021)**	**Gonzalez JE et al. (2021)**	**Ruedisueli I et al. (2023)**	**Arastoo S et al. (2020)**	**Antoniewicz L et al. (2019)**	**Hapstonstall KP et al. (2020)**	**Lyytinen G et al. (2023)**	**Cooke WH et al. (2015)**
**1**	✓	×	×	✓	×	✓	×	×	✓	×	✓	✓
**2**	✓	✓	✓	✓	✓	✓	✓	✓	✓	✓	✓	✓
**3**	×	✓	✓	✓	✓	✓	✓	✓	✓	✓	✓	✓
**4**	✓	✓	✓	✓	✓	✓	✓	✓	✓	✓	✓	✓
**5**	✓	✓	✓	✓	✓	✓	×	✓	×	✓	✓	✓
**6**	✓	✓	✓	✓	✓	✓	✓	✓	✓	✓	✓	✓
**7**	✓	✓	×	×	×	✓	✓	✓	✓	✓	×	×
**8**	×	✓	×	✓	×	×	×	×	×	×	×	×
**9**	×	×	×	✓	×	×	×	×	×	×	×	×
**10**	×	×	✓	×	×	×	×	×	×	×	×	×
**11**	✓	×	✓	✓	×	×	✓	×	✓	✓	✓	✓
**12**	✓	✓	✓	✓	✓	✓	✓	✓	✓	✓	✓	✓
**13**	×	×	×	×	×	×	×	✓	×	✓	×	×
**14**	×	×	×	×	×	×	×	×	×	×	✓	×
**15**	✓	✓	✓	✓	✓	✓	✓	✓	✓	✓	✓	✓
**16**	×	×	×	×	✓	×	✓	✓	✓	✓	✓	×
**17**	✓	✓	✓	✓	✓	✓	✓	✓	✓	✓	✓	✓
**18**	✓	✓	✓	✓	✓	✓	✓	✓	✓	✓	✓	✓
**19**	NI	NI	NI	NI	NI	NI	NI	NI	NI	NI	NI	NI
**20**	✓	✓	✓	✓	✓	✓	✓	✓	✓	✓	✓	✓
**21**	×	✓	×	×	×	×	×	✓	✓	×	✓	×
**22**	✓	✓	✓	✓	✓	✓	✓	✓	✓	✓	✓	✓
**Quality Index**	13/22 (59,09%)	14/22 (63,64%)	13/22 (59,09%)	15/22 (68,18%)	12/22 (54,55%)	13/22 (59,09%)	13/22 (59,09%)	15/22 (68,18%)	15/22 (68,18%)	15/22 (68,18%)	16/22 (72,73%)	13/22 (59,09%)

✓ = meets the item. × = does not meet the item. NI = no information provided.
